# SlideTiler: A dataset creator software for boosting deep learning on histological whole slide images

**DOI:** 10.1016/j.jpi.2023.100356

**Published:** 2023-12-08

**Authors:** Leonardo Barcellona, Lorenzo Nicolè, Rocco Cappellesso, Angelo Paolo Dei Tos, Stefano Ghidoni

**Affiliations:** a Department of Information Engineering, University of Padua, Padua, Italy; b Polytechnic University of Turin, Turin, Italy; c Unit of Pathology and Cytopathology, Ospedale dell’Angelo, Mestre, Italy; d Department of Medicine, DIMED, University of Padua, Padua, Italy; e Department of Integrated diagnostics, Azienda Ospedale-Università, Padua, Italy; f Pathological Anatomy Unit, Padua University-Hospital, Padua, Italy

**Keywords:** Digital pathology, Deep learning, Image preprocessing, Tissue classifier, TCGA

## Abstract

The introduction of deep learning caused a significant breakthrough in digital pathology. Thanks to its capability of mining hidden data patterns in digitised histological slides to resolve diagnostic tasks and extract prognostic and predictive information. However, the high performance achieved in classification tasks depends on the availability of large datasets, whose collection and preprocessing are still time-consuming processes. Therefore, strategies to make these steps more efficient are worth investigation. This work introduces SlideTiler, an open-source software with a user-friendly graphical interface. SlideTiler can manage several image preprocessing phases through an intuitive workflow that does not require specific coding skills. The software was designed to provide direct access to virtual slides, allowing custom tiling of specific regions of interest drawn by the user, tile labelling, quality assessment, and direct export to dataset directories. To illustrate the functions and the scalability of SlideTiler, a deep learning-based classifier was implemented to classify 4 different tumour histotypes available in the TCGA repository. The results demonstrate the effectiveness of SlideTiler in facilitating data preprocessing and promoting accessibility to digitised pathology images for research purposes. Considering the increasing interest in deep learning applications of digital pathology, SlideTiler has a positive impact on this field. Moreover, SlideTiler has been conceived as a dynamic tool in constant evolution, and more updated and efficient versions will be released in the future.

## Introduction

In the era of precision medicine, microscopic examination of tissue morphology remains the cornerstone for clinical decisions and research in oncology.^1^ Pathologists play a crucial role in providing a diagnosis, as well as prognostic and predictive information about a disease by combining data from morphological, phenotypical, ultrastructural, and molecular features.^2^ The digitisation process in pathology laboratories is leading to significant changes, opportunities, and challenges in this field. Thanks to the introduction of digital Whole Slide Images (WSIs), pathologists can now work directly on digital images instead of the classical slides observed under the microscope.^3^

Recent developments in computing sciences, especially in digital image processing, are offering novel tools for advanced WSI analysis.^4^ In particular, Deep Learning (DL) is a breakthrough technology that is having a crucial impact on medical image processing.^5^^,^^6^ DL for WSI analysis is mainly based on Convolutional Neural Networks (CNNs), which apply a hierarchical data processing workflow to autonomously identify local patterns hidden in the images to solve specific tasks, such as tissue classification.^7^^,^^8^ Although CNNs are still the dominant approach in medical image analysis, many recent works started exploiting a new type of neural network named Vision Transformer (VT),^9^ that are able to achieve superior performance thanks to their ability to manage long-range dependencies through a self-attention mechanism.

Despite the great potential of DL and the encouraging results obtained so far in pathology applications, some relevant challenges are still to be overcome. The first challenge in implementing DL models for clinical tasks, such as cancer recognition or cell segmentation, is to create high-quality datasets with an exhaustive representation of the problem to be tackled.^10^ Datasets for training DL networks for classification tasks are composed of images with a label that identifies the category (or class) the image belongs to. Such label identifies one choice among a predetermined set of categories.

The WSI is a gigapixel image that is too large to be used as input for a DL model, and most of the pixels that compose the image are not useful for solving the task.^11^ Unwanted elements, such as tissue artefacts and non-informative background, should be excluded from the training data. To pursue this goal, specific areas within the WSI should be selected and reduced into small annotated tiles that may be used as input data.^12^

Manual processing of WSIs to create annotated datasets is a time-consuming and tedious activity. Currently, several open-access software tools exist for managing preprocessing tasks in digital pathology,^13^ including QuPath,^14^ ASAP,^15^ Histoclean,^16^ QuickAnnotator,^17^ Orbit,^18^ and DSA.^19^ These tools can efficiently handle preprocessing tasks, such as annotation, stain normalisation, colour deconvolution, and segmentation. Most of these tools, such as QuPath, Orbit, and DSA, present an excellent graphical interface also offering a web-based environment (Orbit, DSA). However, a direct and automatised workflow to manage image tiling at different magnification levels is still missing, or requires coding skills. Other tools, such as QuickAnnotator, require Python and Docker environment, hard to be deployed by pathologists or non-IT researchers.

SlideTiler is designed to overcome the limitations of the other available tools thanks to a graphical labelling tool that allows pathologists and biomedical researchers to create large annotated datasets with only few simple steps. The tool is capable of opening slides generated by scanners of many vendors. Using the interactive interface, pathologists can label these images with the help of sophisticated functionalities. After opening the image, the pathologist can draw regions-of-interest (ROIs), namely areas that are going to be labelled. If the user makes a mistake while drawing the ROI, he/she can easily adjust its position and shape. Once the ROI is ready, dedicated options allow setting the dimension of the tiles, the squared region inside a ROI, and the resolution of their content. It is then possible to associate a label to all the tiles inside the region. Labels can be configured by the user depending on the number of different categories that should be distinguished. In the final stage, SlideTiler saves this information together with the data in a hierarchical structure that is easy to exploit for training machine learning algorithms.

**Fig. 1:**
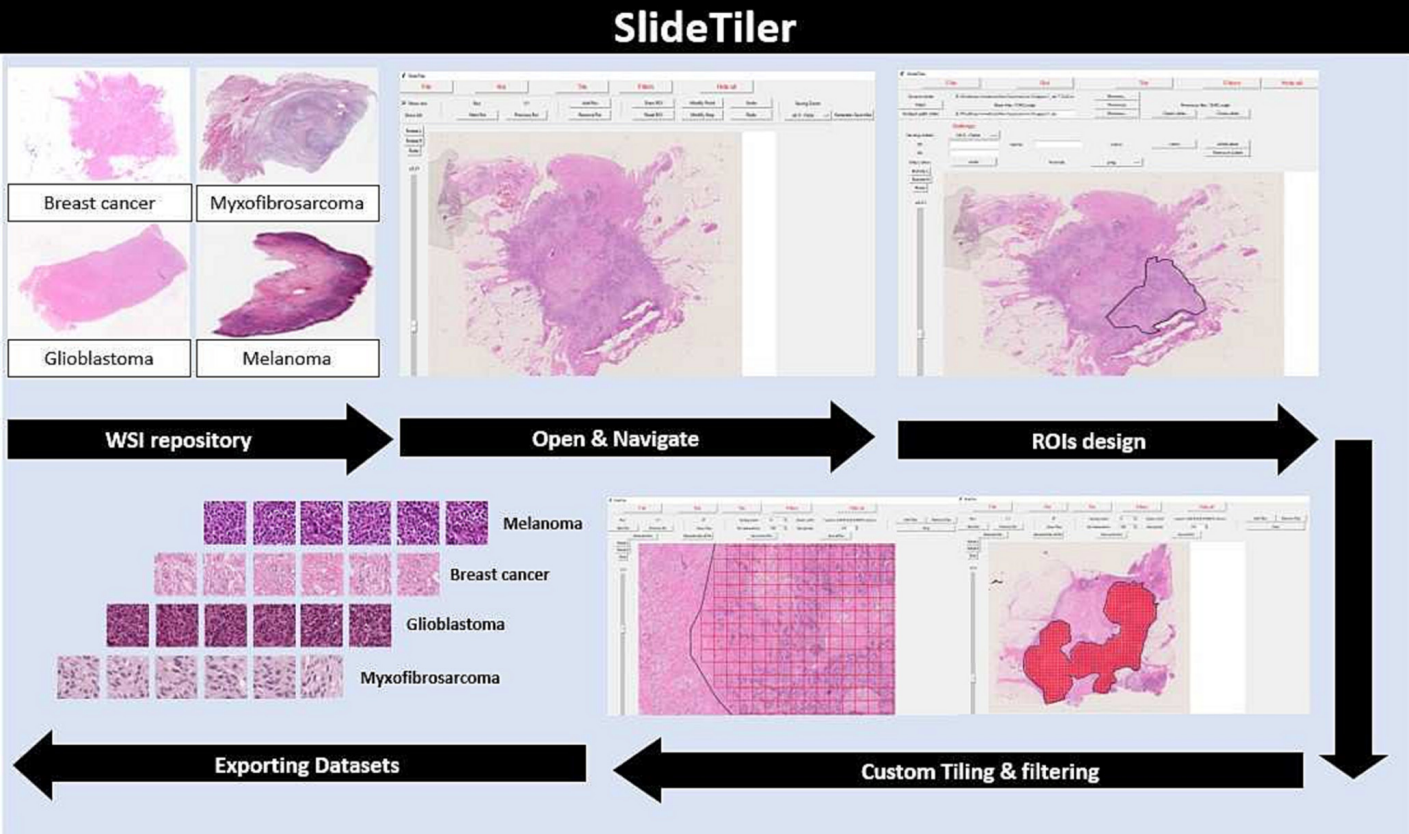
Annotation procedure. SlideTiler can open images from a repository. Using the graphic interface, he/she depicts the region of interest and selects the class. Then, the tiles are generated and filtered. Finally, the tiles are stored along with the annotations.

To demonstrate its scalability, we used SlideTiler to create a dataset with 4 different and clinically relevant tumour histotypes, then used to feed a 4-class classifier to automatically recognise each histotype. The whole workflow is simple, as shown in Fig. 1, and can be fulfilled without specific informatic skills. Thanks to SlideTiler, the creation of datasets is more efficient and accessible to a large number of clinical researchers, who can accurately annotate data. Consequently, more high-quality data will be available, boosting DL models, which accuracy strongly depends on the data used for training.^20^ SlideTiler can also play a pivotal role in clinical and translational applications, in which it can make valuable contributions to the development of digital pipelines for biomarkers discovery to predict patient prognosis and treatment response, and for advanced tissue classification, which is useful for pathologist assistance.^21^

## SlideTiler

### Input and output

**Fig. 2:**
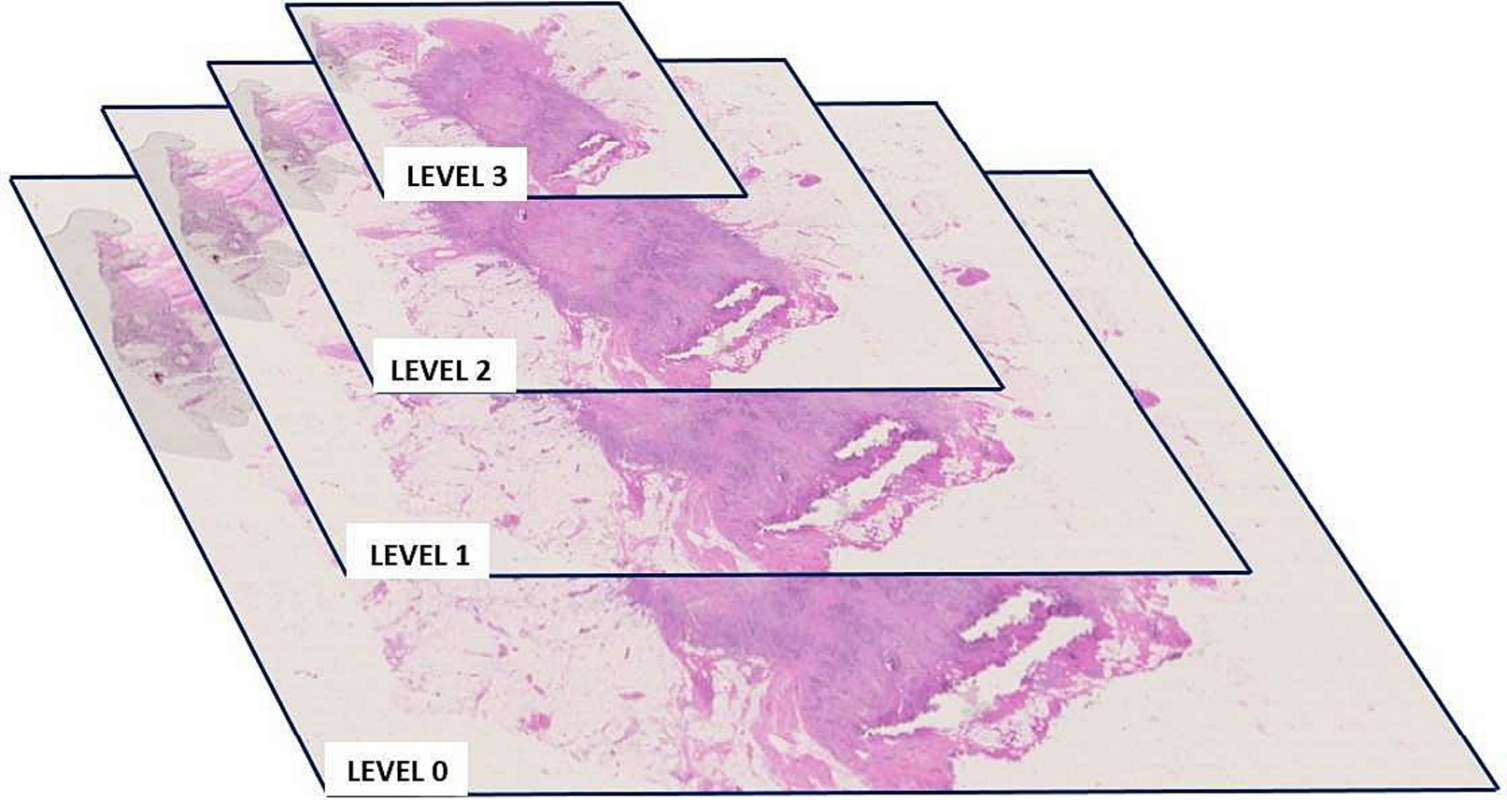
The structure of a whole slide image. The lowest level is the full-resolution image. The higher the level, the lower the resolution.

SlideTiler can visualise WSIs thanks to a graphical user interface and allows users to interact with the image to create annotations. WSIs are organised into multiple copies of the same image at different resolutions, as shown in Fig. 2. Such pyramidal structure is used to efficiently pass from one portion of the image to another because the full-resolution image would use too many computational resources and is difficult to process. The image at the lowest level (level zero) is at full-resolution, whereas at higher levels the resolution is reduced. SlideTiler can manage all the layers of the WSI, allowing users to change the resolution of the annotation and the resolution of the visualised image efficiently. This visualisation of the WSI and the navigation among levels is performed using OpenSlide,^22^ a library offering a shared interface for many scanner vendors and WSI formats: Aperio (.svs,.tif), DICOM (.dcm), Hamamatsu (.vms,.vmu,.ndpi), Leica (.scn), MIRAX (.mrxs), Philips (.tiff), Sakura (.svslide), Trestle (.tif), and Ventana (.bif, .tif). Since pathologists may work with different scanner models, SlideTiler manages all these file types.

**Fig. 3:**
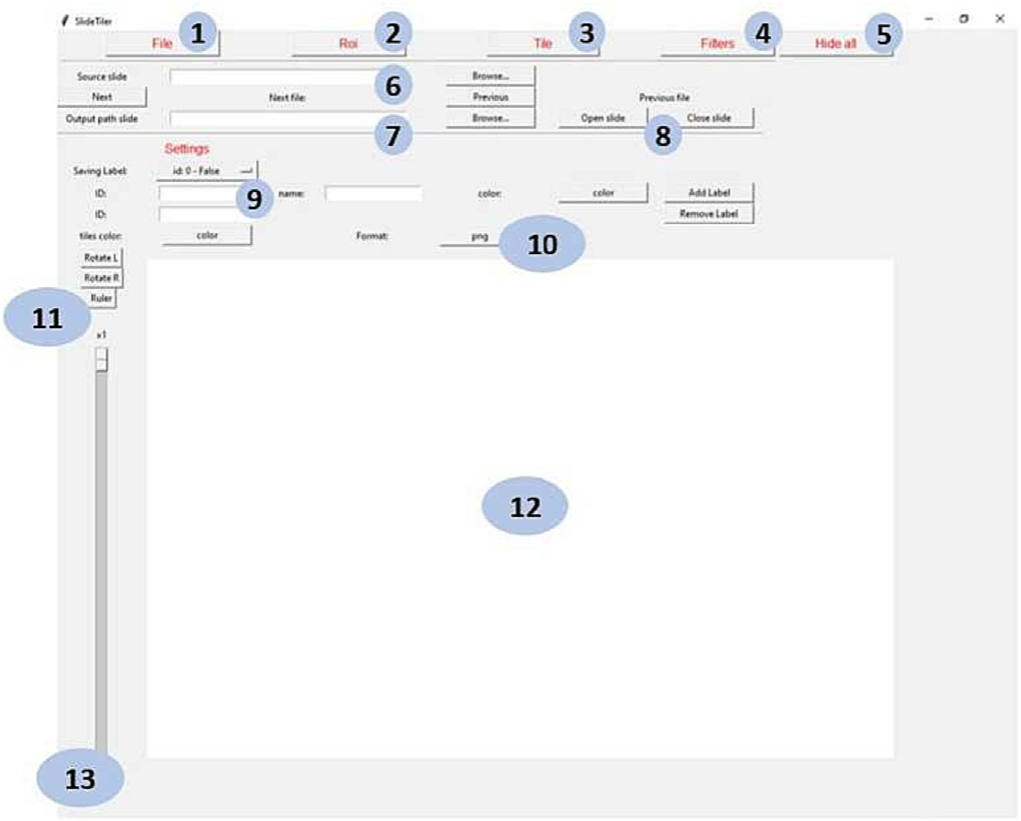
The graphical interface of SlideTiler. Numbers 1–5 are buttons to change the window. For example, for annotating the ROI, the tiles or filtering. Numbers 6 and 7 are the input and output files. Number 8 allows changing files. Number 9 highlight the part where the user can set different label (classes). Number 10 is the output format. Numbers 11 and 13 allow rotating and changing visualisation scale. Number 12 shows the canvas where the slide is shown and where the user may interact to annotate the image.

SlideTiler associates each WSI with the information provided by the pathologist that is working on the image (e.g., defining ROIs) in the form of additional files saved to disk. The tool is also capable of recovering previous annotations, where available, to keep track of what was already processed and avoid duplicating the annotations, since training models using duplicated data with different labels may reduce accuracy. An overview of the main interface of SlideTiler is shown in Fig. 3.

### ROI creation and tiles generation

**Fig. 4:**
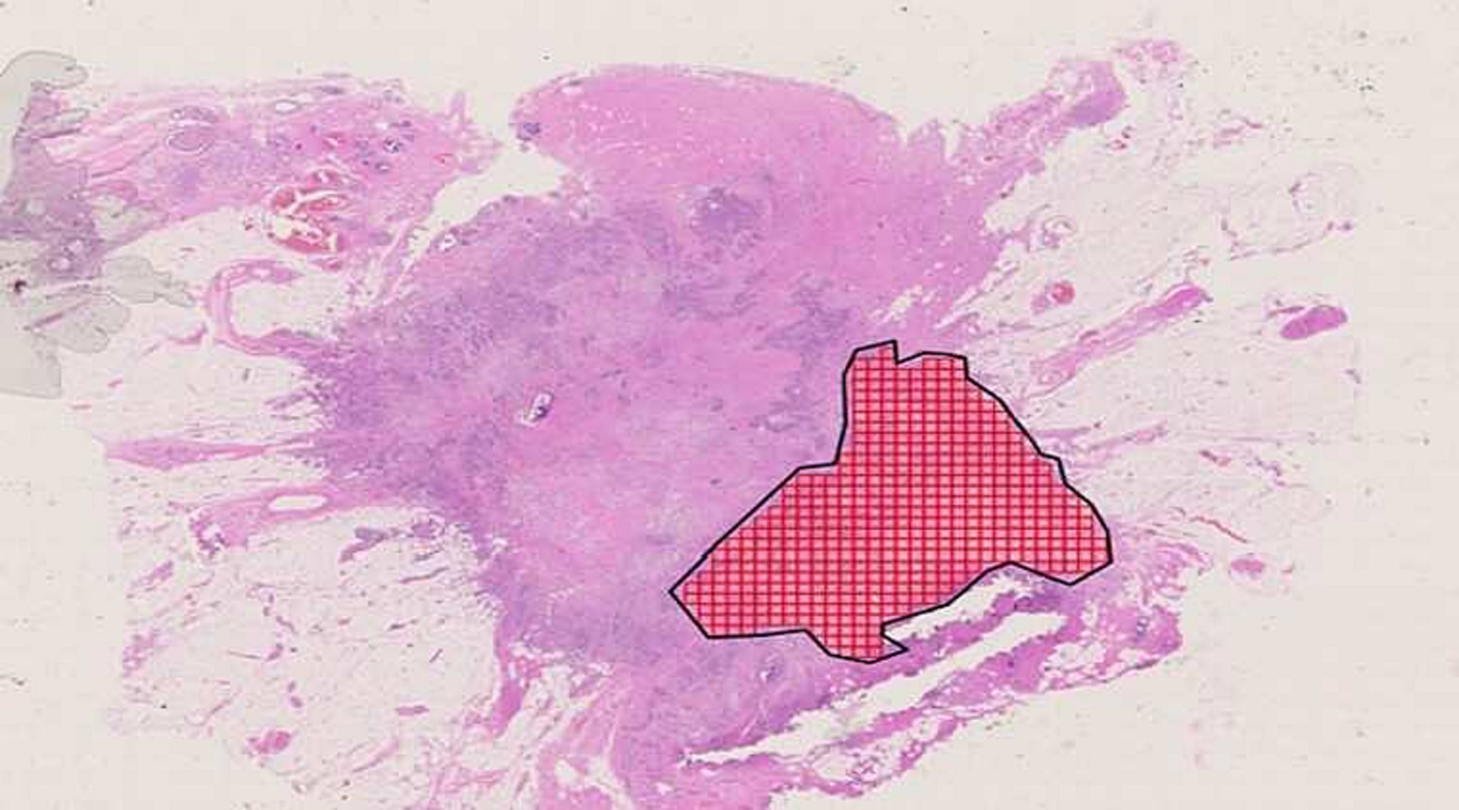
The blue polygon is the ROI, whereas the red squares are the tiles generated inside the ROI.

A ROI is a polygon whose vertices are defined by the user with left clicks. Once the user creates a ROI, this can still be modified in case of mistakes. Tiles are squared regions inside a ROI with a side that is defined in pixels, as shown in Fig. 4, where a ROI and the tiles generated inside it can be seen. SlideTiler can convert the dimensions from pixels to metres to give feedback on the real size of the tiles created. By default, these are referred to level zero (maximum resolution), but the layer can be changed to create tiles at higher levels (lower resolutions).

Depending on user’s preference, tiles are taken either when they are completely included in the ROI, or when a there is a certain degree of overlapping between ROI and tile—the minimum overlap required can be selected by the user. Furthermore, the user can manually remove and add tiles inside the ROI, in case the polygon includes some unwanted areas that should not be saved or missing some relevant portions.

### Saving

Since pathologists may want to annotate different types of cancers or diseases, SlideTiler manages the dynamic setting of the labels: the user can add or remove labels using the intuitive graphical interface.

**Fig. 5:**
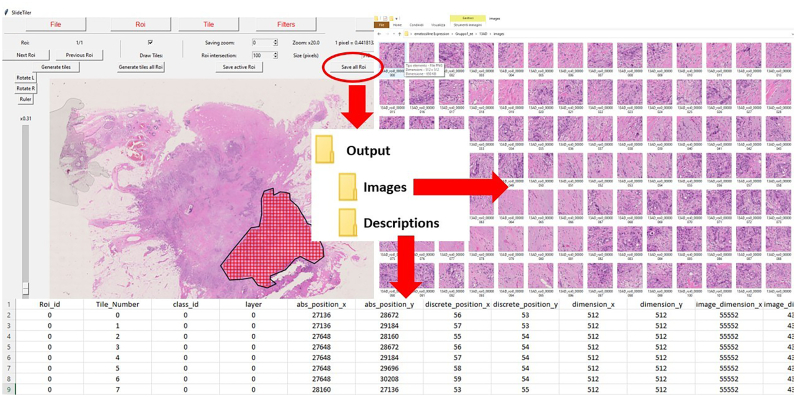
The output structure of SlideTiler. Once the user is satisfied with the annotation, he/she can save it. The tiles are saved in a ‘images; folder and are progressively enumerated. A ‘descriptions’ folder stores the files containing the tile’s characteristics.

When the user saves the tiles, data are stored in a hierarchical structure, as depicted in Fig. 5. The main directory inherits the same name of the slide to keep track of images already labelled. Inside the output directory, data are subdivided into 2 folders: images and descriptions. The former contains all the tiles in the exported format identified with a progressive number, while the latter contains 2 more files in csv format containing information about the ROIs, such as dimension and position, and about each tile, namely label and generating ROI. Tiles can be saved in 2 popular formats: jpeg and png.

### Filters

SlideTiler offers a filtering section that implements intelligent functionalities that help annotate only relevant parts of the image. For example, it incorporates StarDist,^23^ a CNN model that detects and segments cells and nuclei in microscopy images. Thanks to StarDist, the user can set a custom threshold linked to the overall percentage of cell nuclei detected in the tiles—tiles with inadequate cellularity are automatically excluded from the dataset. The filter section is designed in order to facilitate the introduction of more state-of-the-art filters in the future, to improve datasets creation and to facilitate the annotation process.

### Releases

SlideTiler is an open-source software programmed in Python and can be used without restrictions. Moreover, any developer can update or improve its functionalities to adapt the software to new situations or needs. The user interface is created using Tkinter, a Python library for graphical widgets, and Matplotlib.

In addition to having the code open-source, some standalone versions are freely distributed for Windows 10 and 11 operating systems and for Linux systems. These standalone versions do not require any installation, a great advantage for the users without significant informatic skills willing to exploit the software for annotating the WSIs. For Windows, the standalone versions are available as executable files (.exe extension) and its size is about 1.3 GBytes due to the many libraries used to develop the application.

## Experiments and results

### Dataset creation using SlideTiler

To demonstrate the functionalities of SlideTiler, the tool was used to create a dataset suitable for a medical image classification task. Four categories were considered: primary skin melanoma (SKCM), glioblastoma (GBM), breast cancer (BRCA), and high-grade myxofibrosarcoma (MFS). These tumour entities were selected firstly according to the high clinical relevance, and secondly, according to the specific diagnostic expertise of the pathologists involved in this study (SKCM and BRCA: R.C.; GBM and MFS: L.N.). From The Cancer Genome Atlas (TCGA), we selected 399 WSIs including 101 WSIs for MFS; 98 WSIs for BRCA; 100 WSI for GBM and SKCM. Each WSI corresponds to a single patient. Diagnostic haematoxylin & eosin slides for each case were downloaded from the Genomic Data Commons Portal^2^. Before inclusion, all the cases were reviewed by a pathologist—the diagnosis was confirmed according to the latest edition of the corresponding World Health Organisation Classification of Tumours. After data download, a pathologist used SlideTiler to open each slide, drew a ROI including only cancer tissue, and performed ROI tiling to create tiles of size 512×512 pixels at 40× magnification power. The generated tiles were filtered before export to include only tiles with at least 65% of the tile area covered by cell nuclei. To avoid splitting tiles of the same patient among training, validation, and test sets, WSIs of each class were randomly subdivided at patient level to form the training dataset (about 60% of the cases), the validation dataset (about 20%), and the test dataset (about 20%). Overall, 249.462 tiles have been generated: 58.994 tiles for BRCA, 29.764 tiles for GBM, 43.548 tiles for MFS, and 117.156 tiles for MM.

It should be observed that the dataset was obtained from 399 WSIs generating 249.462 tiles—an average of 625 tiles per slide. However, it should be pointed out that tiles taken from the same WSI have a high level of correlation, which might interfere with the training process. To reduce correlation within the dataset, in the following experiments the training and validation datasets were sub-sampled: the training set contains 2951, 2994, 2772, and 2840 randomly selected tiles for BRCA, GBM, MFS, and SKCM, respectively, and the validations set 1001, 973, 1001, and 951 tiles for BRCA, GBM, MFS, and SKCM. However, it is worth highlighting that all the test patients’ tiles have been used for the evaluation: 10 942 from 21 patients for BRCA, 5864 tiles from 21 patients for GBM, 9882 from 19 patients for MFS, and 27 552 tiles from 20 patients for SKCM.

### Training of CNN and transformer models

The dataset was used for training state-of-the-art models for image classification. We tested 5 different models: 4 Convolutional Neural Networks, namely Resnet 50,^24^ Resnet 101,^24^ EfficientNet B4,^25^ and VGG 16^26^ and a Vision Transformer model, DeiT.^27^ We implemented these models using PyTorch and the Timm library.^28^ For each model, we started from the network pretrained on the ImageNet dataset^29^; the Adam optimizer and the cross-entropy loss were employed for training.

The networks were trained varying a number of hyperparameters, namely: learning rate, batch size, image size, and augmentation. Image size was varied considering tiles having different numbers of pixels but covering the same portion of the WSI, which means modifying the resolution. Augmentation was perfomed randomly modifying gaussian blur, rotations and flips, and image contrast, saturation, brightness, and hue. The hue-shift is a technique for uniformly switching the hue of every pixel in the image—this causes a shift in colour, which is useful to obtain a network that can successfully cope with tiles prepared using multiple colouring techniques. Combinations of the augmentation factors were also considered: *mix1* is the mixture of random rotations with blur filter, *mix2* adds bright and contrast augmentations to *mix1*, and *mix3* is the combination of all the augmentations considered. The best hyperparameters were chosen based on a grid search approach: the models were trained with every possible combination of the hyperparameters among the values reported in Table 1.

**Table 1:** Hyperparameters for the grid search. For the augmentation, *mix1* is rotations with blur, *mix2* adds bright and contrast augmentations to *mix1*. Finally, *mix3* is the combination of all the augmentations considered.

Parameter	Values
Learning rate	$1\times 10^{-3}$, $5\times 10^{-4}$, $1\times 10^{-4}$, $5\times 10^{-5}$, $1\times 10^{-5}$
Batch size	32, 64
Image size	64×64, 128×128, 224×224
Augmentation	Random gaussian blur, random rotations and flips, random contrast, random saturation, random brightness, random hue-shift, *mix1*, *mix2*, *mix3*

The training dataset contains an enormous number of tiles. Consequently, the parameters of the model change drastically after each epoch, making the early stopping unable to prevent overfitting. If we wait until the end of the entire epoch to stop the training, the model may end in a highly sub-optimal solution. To cope with this problem and avoid overfitting, we performed intra-training validation steps: the models were validated 8 times for each training step, meaning that we stopped each epoch before the end to check the effect on the validation dataset, applying early stopping after 5 consecutive validation steps without improvements.

## Results

For the experiments, we report the following metrics: accuracy, sensitivity, specificity, and F1-score. The accuracy is the percentage of correctly classified in the entire dataset. The sensitivity is the ratio between correctly predicted positive and the total number of actual positive instances. The specificity is the ratio of true-negative predictions to the total number of actual negative instances. A higher sensitivity score indicates a higher ability to detect positive instances accurately, whereas a higher value of specificity indicates a higher accuracy in identifying negative cases. The F1-score is the harmonic mean of sensitivity and precision, where the precision is the proportion of true-positive predictions compared to all positive predictions made by the model. All the metrics are computed on each single tile. For each disease, sensitivity and specificity are calculated considering other classes as negative samples. Consequently, the sensitivity is the probability of detecting a positive result conditioned on positive samples, whereas the specificity considers the negative samples.

**Fig. 6:**
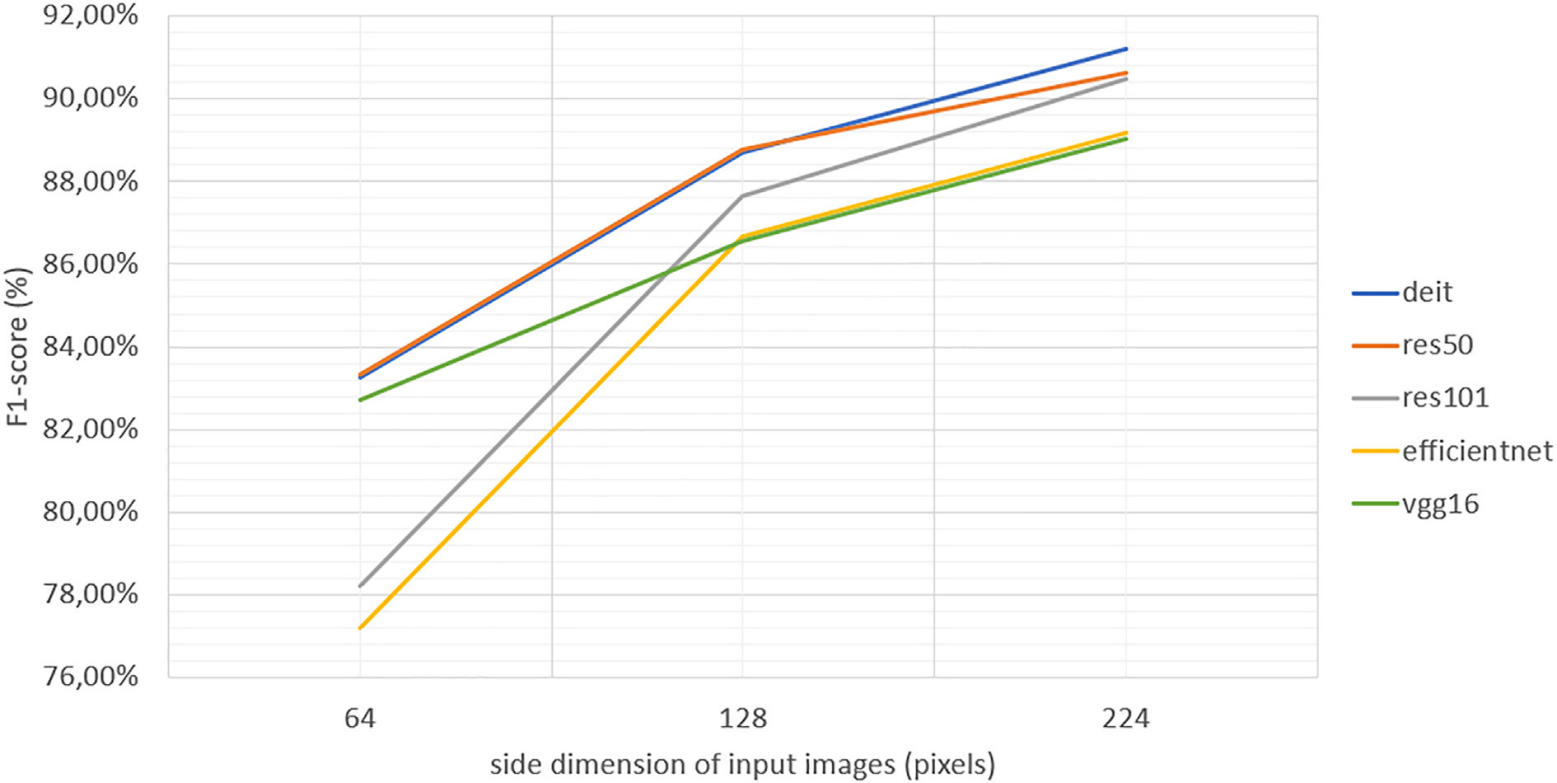
F1-Score correlated to the input image size. Increasing the size of the input image brings positive effects for each model tested.

As previously mentioned, we adopted a grid-search approach: the best model was chosen based on the highest F1-score. Table 2 shows the results in the test dataset. The best-performing model was found to be DeiT,^27^ which exploits the self-attention mechanism to relate different parts of the tiles to make the final prediction. It is easy to notice that all the models reported in the table share the same image size (224×224 pixels), meaning that a higher resolution helps extract relevant features for the final classification. This hypothesis is confirmed by Fig. 6, where the F1-score obtained by each model increases by increasing the input tile size, the maximum value being limited by memory constraints of the GPU (NVidia GeForce GTX 2080 Ti with 12 GByte). We also evaluated the effect of the augmentations used in the grid search approach (Table 1) by selecting the best model for each augmentation and tile size. We noticed that the augmentation where the hue was changed obtained scarce results. Table 3 proves the effect by reporting the best results obtained and the results using the hue augmentation for each image size. Besides the decrease in performance due to the hue-shift, no other significant patterns were visible. The best augmentation depends on the model and the input size.

**Table 2:** Results of the 5 models. The table reports the accuracy, F1-score, the sensitivity, and specificity. The hyperparameters are also reported: LR is the learning rate of the optimiser, BS is the batch size used during training, Img size is the dimension of the squared input image, and augmentation reports the type of data augmentation done during training. The bold represents the highest value.

Model	Img size	LR	BS	Augmentation	Accuracy	F1-score	Sensitivity	Specificity
VGG16^26^	224×224	$1\times 10^{-4}$	32	Saturation	89.08	89.03	87.09	94.88
ResNet 50^24^	224×224	$1\times 10^{-4}$	64	mix1	90.99	90.61	90.89	96.30
ResNet 101^24^	224×224	$5\times 10^{-4}$	32	Rotations and flips	90.91	90.49	89.51	96.11
EfficientNet B4^25^	224×224	$1\times 10^{-3}$	32	mix2	89.82	89.18	88.86	95.55
DeiT^27^	224×224	$5\times 10^{-5}$	32	Contrast	**91.43**	**91.22**	**91.24**	**96.39**

**Table 3:** Comparison of hue-shift augmentation and the best augmentation in terms of F1-score. Hue-shift is always highly reducing the F1-score, meaning that changing the real colour components of the image reduces performance.

Image size	Augmentation	VGG 16^26^	ResNet 50^24^	ResNet 101^24^	EfficientNet B4^25^	DeiT^27^
64×64	Best	82.71	83.35	78.97	77.19	83.27
64×64	Hue	78.40	77.62	75.04	71.24	80.82
64×64	Difference	-4.30	-5.73	-3.93	-5.95	-2.45
128×128	Best	86.56	88.79	87.65	86.67	88.71
128×128	Hue	82.06	86.56	78.87	81.16	85.43
128×128	Difference	-4.49	-2.23	-8.78	-5.51	-3.28
224×224	Best	89.03	90.61	90.49	89.18	91.22
224×224	Hue	80.12	86.19	83.58	84.91	84.87
224×224	Difference	-8.92	-4.43	-6.91	-4.28	-6.35

**Fig. 7:**
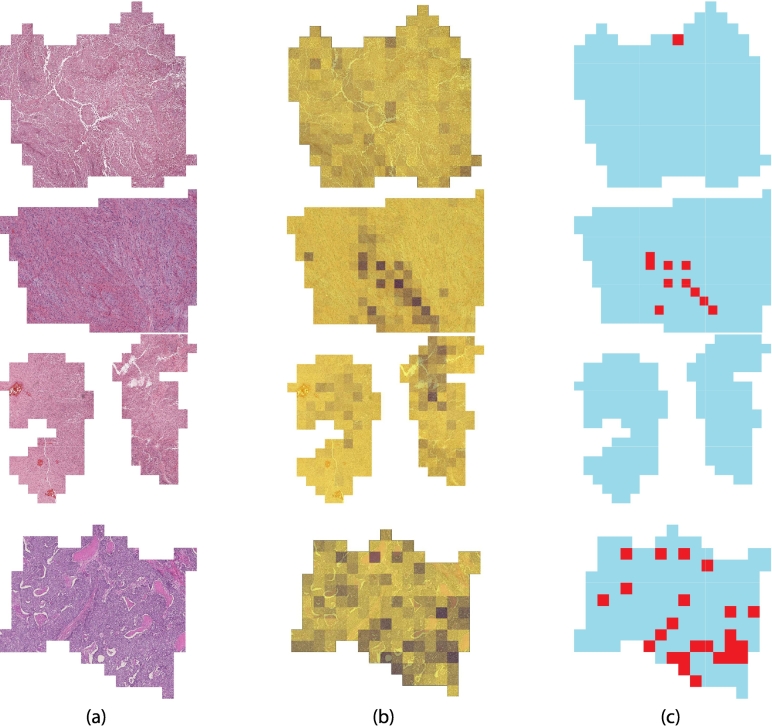
Examples of predictions on WSIs—(a) shows the real image, (b) the probability of the correct class, and (c) the correctly predicted labels. In the latter, using a majority voting is possible to retrieve the correct label (in cyan) even if some tiles are wrongly predicted (in red). Each row shows a different class: skin melanoma (SKCM), high-grade myxofibrosarcoma (MFS), glioblastoma (GBM), and breast cancer (BRCA).

We then evaluated the results considering the whole slide images instead of the single tiles. For computing the final prediction, we merged the classification of the patient’s tiles with a majority voting scheme. Fig. 7 shows some examples of predictions on WSIs. The WSI is divided into tiles, each one is then separately classified by the models. Thanks to the high accuracy obtained on single tiles, almost every patient is correctly classified. A curious aspect emerged from the results reported in Table 4: DeiT^27^ was not confirmed as the best model. Instead, EfficieNet^25^ obtained the best results in all metrics.

**Table 4:** Results of the 5 models. The table reports the accuracy, F1-score, the sensitivity, and specificity. The hyperparameters are also reported: LR is the learning rate of the optimiser, BS is the batch size used during training, Img size is the dimension of the squared input image, and augmentation reports the type of data augmentation done during training. The bold represents the highest value.

Model	Img size	LR	BS	Augmentation	Accuracy	F1-score	sensitivity	specificity
VGG16^26^	224×224	$1\times 10^{-4}$	32	Saturation	95.06	95.12	94.99	98.36
ResNet 50^24^	224×224	$1\times 10^{-4}$	64	mix1	95.06	95.05	94.99	98.35
ResNet 101^24^	224×224	$5\times 10^{-4}$	32	Rotations and flips	93.83	93.98	93.99	97.94
EfficientNet B4^25^	224×224	$1\times 10^{-3}$	32	mix2	**98.77**	**98.78**	**98.81**	**99.59**
DeiT^27^	224×224	$5\times 10^{-5}$	32	Contrast	95.06	95.14	95.05	98.35

The deep learning models presented so far can effectively support pathologists during the diagnosis thanks to the high performance level achieved. This was possible thanks to the large training dataset, that was easily generated thanks to SlideTiler, that allowed pathologists to extract annotations of WSIs.

## Discussion

The increasing use of AI is driving major advancements in healthcare.^30^ By introducing SlideTiler, we proposed a tool to address the challenge of creating high-quality datasets from WSIs. SlideTiler offers a simple and direct way to generate accurate datasets with no limitation in class numbers, and managing, through a simple and intuitive graphical user interface, ROIs shape, tiles dimension, tissue magnification level and tiles filtering according to cellularity.

Comparing SlideTiler against other available tools (see Table 5), we find that it shares most of the key characteristics, but allows exporting tiles without the need for custom code implementation. This aspect is crucial for creating a tool that is manageable and accessible for every pathologist regardless of their background in informatics. In this study, 2 pathologists used SlideTiler directly on their PC, exploiting the standalone version available for Windows 10 without installing the software, thus exploiting a major strength of the software. SlideTiler managed about 400 different WSIs and the users did not report any problem. WSIs of each class were stored in the same folder because Slidetiler allows users to open or close slides directly from the file management window, increasing the productivity of the annotation process. The procedure to generate tiles for each class of cancer was entirely managed by 2 pathologists without the need for software developers, highlighting the scalability of SlideTiler within a clinical context.

**Table 5:** Comparison of SlideTiler against other open-source programs. For each tool, we report several features: (i) it is available open-source; (ii) the precompiled executable is available for download (.EXE); (iii) it enables WSI navigation; (iv) it enables the user to annotate areas of the WSI; (v) it provides an option for dividing areas of WSI into tiles; (vi) it lets the user export the tiles; (vii) the tool provides direct access to machine/deep learning models. In the table, (*) identifies the tools that require custom code implementation to export tiles.

Tool	Open-source	.EXE	WSI navigation	Annotation	Tiling	Tiles exporting	Direct analysis
ASAP^15^	Yes	Yes	Yes	Yes	No	No	No
QuickAnnotator^17^	Yes	No	Yes	Yes	No	No	No
DSA^19^	Yes	No	Yes	Yes	No	No	No
Qupath^14^	Yes	Yes	Yes	Yes	Yes	No*	Yes
Orbit^18^	Yes	Yes	Yes	Yes	No	No	Yes
SlideTiler	Yes	Yes	Yes	Yes	Yes	Yes	No

Experiments focused on a 4-class deep-classifier trained and tested using tiles generated by SlideTiler. The tiles were used to feed 4 different CNN models commonly used to solve histopathological classification tasks (ResNet50, ResNet101, EfficientB4, and VGG16), and a transformer model (DeiT). The latter showed higher accuracy in the single tile classification. However, in the whole slide image prediction task through majority voting, EfficientNet obtained better results than DeiT. The results with single tiles indicate that DeiT is the model with the highest capability of learning the task, but the majority voting may not be the best approach for such models. Although there are no exhaustive studies that compare performances between CNN-based systems and VTs, studies on medical imaging reported better results for VTs.^9^ In this work, we confirm such hypothesis also for the tiles classification into primary skin melanoma, glioblastoma, breast cancer, and high-grade myxofibrosarcoma.

We hypothesise that the DeiT model is more capable of exploiting long-range connections in images and is less related to colour patterns, following the studies on VT models.^31^^,^^32^^,^^33^ These characteristics are particularly relevant in histological images, in which the features reflecting the biology of the underlying disease may depend on complex architectural characteristics of the tissue rather than single image objects as single cells.

## Conclusions

Artificial intelligence is playing a major role in revolutionising the way diseases are diagnosed and treated. However, the success of AI and data-driven technologies largely depends on the quality of the data used to train them. To achieve high accuracy and a strong generalisation, it is crucial to ensure that input data is of the highest quality.

In this paper, we addressed the challenge of creating high-quality datasets for pathological research, specifically for DL analysis of digital WSIs. We introduced a graphical tool called SlideTiler that enables pathologists and researchers to quickly and easily create large annotated datasets without requiring specific informatic skills and in few simple steps.

SlideTiler represents a valuable tool for creating annotated datasets, as demonstrated by the experiments reported. However, some limitations and critical points are still recognised, opening the way to future developments. First, the software is only compatible with Windows 10, 11, and Linux, which limits its accessibility to researchers who use other operating systems. Moreover, the current downloadable executable file for SlideTiler occupies a large amount of space, making it inconvenient for users with limited storage capacity. A solution to these problems is offering a cloud-based version of the software with a web-based interface, which does not require the user to download and store the application locally. Second, the software currently has limited capabilities for automatically discarding tiles with artefacts, which could interfere with the analysis. This is a time-consuming task for users, as it must be done by scrolling through the individual tiles. A solution to this limitation is integrating into SlideTiler additional filters that can classify tiles and automatically remove those affected by artefacts. Third, SlideTiler only opens one slide at a time, which is a limitation for researchers who need to compare multiple slides simultaneously. Further releases will integrate a feature that allows users to open and view multiple slides simultaneously in different windows or tabs.

## Declaration of Competing Interest

The authors declare that they have no known competing financial interests or personal relationships that could have appeared to influence the work reported in this paper.

The following are the supplementary data related to this article.

Supplementary video 1 Supplementary video 1

Supplementary data to this article can be found online at https://doi.org/10.1016/j.jpi.2023.100356.

## Data Availability

We will release the tool on github: https://github.com/leobarcellona/SlideTiler.

## Footnotes

2 https://portal.gdc.cancer.gov.
